# ERK-mediated TIMELESS expression suppresses G2/M arrest in colon cancer cells

**DOI:** 10.1371/journal.pone.0209224

**Published:** 2019-01-10

**Authors:** Beth K. Neilsen, Danielle E. Frodyma, Jamie L. McCall, Kurt W. Fisher, Robert E. Lewis

**Affiliations:** 1 Eppley Institute, Fred & Pamela Buffett Cancer Center, University of Nebraska Medical Center, Omaha, Nebraska, United States of America; 2 Department of Pathology and Microbiology, University of Nebraska Medical Center, Omaha, Nebraska, United States of America; University of South Alabama Mitchell Cancer Institute, UNITED STATES

## Abstract

The cell cycle is under circadian regulation. Oncogenes can dysregulate circadian-regulated genes to disrupt the cell cycle, promoting tumor cell proliferation. As a regulator of G2/M arrest in response to DNA damage, the circadian gene Timeless Circadian Clock (TIMELESS) coordinates this connection and is a potential locus for oncogenic manipulation. TIMELESS expression was evaluated using RNASeq data from TCGA and by RT-qPCR and western blot analysis in a panel of colon cancer cell lines. TIMELESS expression following ERK inhibition was examined via western blot. Cell metabolic capacity, propidium iodide, and CFSE staining were used to evaluate the effect of TIMELESS depletion on colon cancer cell survival and proliferation. Cell metabolic capacity following TIMELESS depletion in combination with Wee1 or CHK1 inhibition was assessed. TIMELESS is overexpressed in cancer and required for increased cancer cell proliferation. ERK activation promotes TIMELESS expression. TIMELESS depletion increases γH2AX, a marker of DNA damage, and triggers G2/M arrest via increased CHK1 and CDK1 phosphorylation. TIMELESS depletion in combination with Wee1 or CHK1 inhibition causes an additive decrease in cancer cell metabolic capacity with limited effects in non-transformed human colon epithelial cells. **The data show that** ERK activation contributes to the overexpression of TIMELESS in cancer. Depletion of TIMELESS increases γH2AX and causes G2/M arrest, limiting cell proliferation. These results demonstrate a role for TIMELESS in cancer and encourage further examination of the link between circadian rhythm dysregulation and cancer cell proliferation.

## Introduction

Several studies have demonstrated circadian rhythms are dysregulated in cancer cells [1, 2]. This dysregulation can be a result of aberrant oncogenic signaling as oncogenes can drive the expression of circadian genes effectively hijacking the circadian cycle. MYC drives the expression of REV-ERBα, which decreases BMAL1 expression releasing its tumor suppressive effects and altering cell metabolism [3]. Recent work has also shown that restoring circadian rhythmicity *in vitro* decreases proliferation of cancer cells, and circadian dosing of certain chemotherapeutics increases their efficacy [4]. Large studies have correlated shift work and altered sleep/wake patterns with an increased risk of cancer [5–9]. This suggests circadian rhythm dysregulation is not merely a downstream effect of oncogenic signaling, but plays a pro-tumorigenic role.

Independent of the current literature suggesting that circadian dysregulation promotes cancer, we used Functional Signature Ontology (FUSION) [16–18], which is an unbiased approach to screen for functionally-related genes that are selectively required for colon cancer cell survival, but likely dispensable for normal cells. This analysis identified three circadian genes, one of which was Timeless Circadian Clock (TIMELESS), a lesser-known circadian gene that interacts with both Cryptochrome (CRY) and Period (PER) proteins and acts on the negative arm of the circadian cycle.

In Drosophila, TIMELESS regulates the circadian rhythm by physically interacting with PER to negatively regulate CYC/CLOCK. In mammals, however, TIMELESS has an expanded functional role in cells. TIMELESS has been shown to promote DNA replication and DNA damage repair [19–27], stabilize the replication fork [19, 21, 23], support telomere maintenance [22, 28], and is essential for embryonic development [29–32].

Recent studies have shown that the cell cycle is under circadian regulation [33–36] and TIMELESS could be the mediator that coordinates this connection [25]. TIMELESS expression is regulated both by the circadian rhythm and cell cycle with the highest expression occurring at night and during S and G2 phases in normal human fibroblasts, respectively [25]. TIMELESS depletion has been shown to limit the ability of cells to trigger DNA damage-associated checkpoint arrest at intra-S checkpoints and G2/M checkpoints [25, 37, 38], which could further sensitize cells to DNA damaging agents [37]. Loss of TIMELESS also caused defects in mitotic progression [23, 28] because TIMELESS synchronizes replication termination and subsequent mitotic kinase (CDK1, Auroras A and B, PLK1) activation [28].

Examining the expression of circadian genes in cancer has incidentally revealed that TIMELESS is frequently overexpressed in breast cancer [10–12], colon cancer [13], lung cancer [14], and cervical cancer [15].Based on the overexpression of TIMELESS in cancer and its described mechanisms of action that could support high fidelity and rapid DNA synthesis, it is likely TIMELESS plays a role in maintaining aberrant cancer cell proliferation.

This study demonstrates that TIMELESS is overexpressed in colon cancer cells at least in part due to increased ERK signaling in cancer. Additionally, TIMELESS depletion slows cancer cell proliferation by inducing G2/M arrest as a result of DNA damage triggering inactivating phosphorylation of CDK1. The combination of TIMELESS depletion with Wee1 or CHK1 inhibition demonstrates additive detrimental effects on colon cancer cells.

## Materials and methods

### Analysis of TCGA data

mRNA expression was analyzed based on the FPKM-UQ normalized RNA-Seq values of normal solid tissue samples and primary tumors from within The Cancer Genome Atlas (TCGA) Breast Invasive Carcinoma (BRCA)(Number of Samples from Normal N = 113 and Primary Tumor N = 1102), Colon Adenocarcinoma (COAD) (N = 41 and N = 478), Lung Adenocarcinoma (LUAD)(N = 59 and N = 533), Lung Squamous Cell Carcinoma (LUSC)(N = 49 and N = 502), Glioblastoma (GBM)(N = 5 and N = 156), Prostate Adenocarcinoma (PRAD)(N = 93 and N = 498), Pancreatic Ductal Adenocarcinoma (PAAD)(N = 4 and N = 177), Sarcoma (SARC)(N = 2 and N = 259) datasets and primary tumors only from Ovarian Serous Cystadenocarcinoma (OV)(N = 374) and Cervical Squamous Cell Carcinoma (CESC)(N = 304) datasets.

### Cell culture

Colorectal cancer cell lines HCT116, LoVo, RKO, HCT15, SW480, SW620, and T84 were purchased directly from American Type Culture Collection (ATCC). Cells were grown in Dulbecco’s Modified Eagle’s Medium containing high glucose and L-glutamine with 10% fetal bovine serum. All colorectal cancer cells were grown at 37 °C with ambient O_2_ and 5% CO_2_. Immortalized non-transformed human colonic epithelial cell lines (HCEC) and HCEC exogenously expressing G12V mutant H-Ras (HCECs + Ras) were a gift from J. Shay (UT Southwestern) [39]. HCECs were grown in medium composed of 4 parts DMEM to 1 part media 199 (Sigma-Aldrich) with 2% cosmic calf serum (GE Healthcare), 25 ng/mL EGF, 1 μg/mL hydrocortisone, 10 μg/mL insulin, 2 μg/mL transferrin, and 5 nM sodium selenite. HCECs were grown in a hypoxia chamber with 2% O_2_ and 5% CO_2_ at 37 °C.

### RT-qPCR

RNA was harvested using 0.5 mL TriReagent (MRC, TR118) and stored at -80 °C until extraction. RNA was extracted per manufacturer’s protocol and final RNA was eluted with nuclease-free water. RNA was quantified using the NanoDrop 2000 (Thermo Scientific). Reverse transcription was performed using iScript Reverse Transcription Supermix for RT-qPCR (Bio-Rad, 170–8840) with 1 μg of total RNA per 20 μL reaction. RT-qPCR was performed using the primers and conditions listed in S1 Table. All targets were amplified using SsoAdvanced Universal SYBR Green Supermix (Bio-Rad) with 40 cycles of a 2-step program (95 °C x 5 sec, T_m_ x 45 sec).

An R script was generated to implement the qBase analysis as described in in the qBase methods paper by J. Hellemans *et al*.[40] with the following modifications to correct typographical errors in the original publication. In formula 4, a sqrt was added around (h-1). In formula 5, a negative sign was added before the 1/slope exponential factor. In formula 12, SD was replaced with SE. Two housekeeping genes, HPRT and GAPDH were included for normalization. All primers were validated using a standard curve to quantify efficiency. Efficiency had to be between 1.9 and 2.1 in order for primers to be considered validated and was used in mRNA quantification. Melting curves were visually inspected to ensure appropriate amplification occurred in each experiment. Each mRNA quantification was obtained from three independent samples for each cell line. mRNA was quantified in technical triplicate for each independently collected sample to ensure accurate and precise quantification was obtained.

### Western blot analyses

Whole cell lysate extracts were prepared in radioimmunoprecipitation assay (RIPA) buffer comprised of 50 mM Tris-HCl, 1% NP-40, 0.5% Na deoxycholate, 0.1% Na dodecyl sulfate, 150 mM NaCl, 2mM EDTA, 10 mM NaF, 10 μg/mL aprotinin, 10 μg/mL leupeptin, 2 mM EGTA, 2 mM PMSF, 0.5 mM Na_3_VO_4_, and 10 mM NaPPi. Protein concentration was determined using the Promega BCA protein assay. SDS-PAGE was performed, nitrocellulose membranes were blocked in Odyssey PBS blocking buffer (LI-COR Biosciences, 927–40000) for at least 45 minutes, and incubated in primary antibody (listed below) overnight at 4 °C. LI-COR fluorescent secondary antibodies (IRDye 800CW, 680LT, or 680RD) were diluted 1:10,000–1:50,000 in 0.1% TBS-Tween (for nitrocellulose). Membranes were imaged using the LI-COR Odyssey Scanner that only records a useable digital image if the signals are within the linear range of its detection. If an area is overexposed such that the band is not visually representative of the expression and cannot be quantified, the image contained either bright red or bright cyan blue pixels and densitometry analysis returned a value of infinity. Therefore, if the exposure of a western blot was outside the linear range, the blot was reimaged using decreased intensity settings. The digital image was then analyzed using the Li-Cor Image Studio Lite software via densitometry to quantify the signal of each band. This process was done for both the target protein and loading controls in each lane. The ratio of the target protein to the loading control (tubulin or actin) was then compared between experimental conditions to quantify the effect of treatment.

### Antibodies

Primary antibodies were diluted as follows: β-Actin (C-4, 47778, Santa Cruz) 1:2000; P-CDK1 (Y15)(9111, Cell Signaling) 1:1000; T-CDK1 (77055 and 9112, Cell Signaling) 1:1000; P-CHK1 (S345)(2348, Cell Signaling) 1:1000; T-CHK1 (G-4, 8408, Santa Cruz) 1:1000; P-ERK (9106, Cell Signaling) 1:1000; ERK (9102, Cell Signaling) 1:1000; Phospho-Histone H2A.X (Ser139)(γH2AX)(2577, Cell Signaling) 1:1000; H2A.X (2595, Cell Signaling) 1:1000; PARP (9542, Cell Signaling) 1:1000; Ras (sc-30, Santa Cruz) 1:1000; P-RSK (S380)(9341, Cell Signaling) 1:1000; T-RSK (601225, BD Biosciences) 1:1000; TIMELESS (A300-961A, Bethyl) 1:5000; and α-tubulin (B-5-1-2, Santa Cruz) 1:2500.

### siRNA reverse transfections

The smartPool of four siRNA oligos (Dharmacon) targeting TIMELESS or ERK1/2 were used for RNAi-mediated target depletion unless otherwise specified. Pooled (smartPool) or individual (S2 Table) ON-TARGET plus siRNAs (DharmaconGE) were introduced into the cell lines listed above following the Lipofectamine RNAiMAX (Invitrogen) reverse transfection protocol and as described following: 5 μL of RNAiMax was added to 2 mL of cells in normal culture media (150,000 cells/mL), 500 uL Opti-MEM media in 6-well plates with a final RNAi concentration of 40 nM. HCECs were transfected following the RNAiMax reverse transfection protocol using 5 μL RNAiMax transfection reagent per 3–5 mL of media and 100,000 cells/mL with a final RNAi concentration of 20 nM in 6 cm plates (CorningTM, PrimariaTM) or on 6-well plates. After a 72-hour transfection, cells were lysed in RIPA lysis buffer with protease and phosphatase inhibitors as described in the Western Blot Analyses section.

### Anchorage-independent growth on poly-2-hydroxyethyl methacrylate (polyHEMA)-coated plates

10 mg/ml polyHEMA stock solution was made by dissolving polyHEMA in 95% ethanol and shaking at 37 °C until fully dissolved (6 hours to overnight). 96-well plates were coated with polyHEMA by evaporating 100–200 μl of the 10 mg/ml stock polyHEMA solution in each well. Cells were plated in complete medium on polyHEMA-coated wells at a concentration of 1.5–2 x 10^4^ cells/100 μl 48 hours post-transfection (as described above). Cell metabolic capacity was measured in relative light units (RLU) per the manufacturers’ protocol using the CellTiter-Glo Luminescent Cell Viability Assay (Promega). Specifically, this was done by adding 90 μl of CellTiter-Glo reagent, shaking for two minutes to lyse the cells, incubating at room temperature for 10 minutes, and measuring luminescence (POLARstar OPTIMA).

### Cell metabolic capacity assay

5,000 (or 10,000 for HCEC) cells/well were transfected on white or clear 96-well plates. Transfections were done as described above but at a ratio of 1:25 for all of the reagents. For combination TIMELESS siRNA transfection and Wee1 or CHK1 inhibition, DMSO, 300 nM of MK-1775 or 300 nM of AZD7762 was added 48 hours after transfection. At 72 or 96 (started with half as many cells) hours post-transfection, 10 μL of alamarBlue (ThermoFisher Scientific) was added to each well (100 μL) for drug treatment experiments or 100 μL of alamarBlue was added per mL of media and media was replaced for transfection-only experiments. Plates were incubated at 37 °C for 1–3 hours and fluorescence was measured (POLARstar OPTIMA). Results were background subtracted (well with media + alamarBlue without any cells) and normalized to the control wells. In other instances, cell metabolic capacity was measured per the manufacturers’ protocol using the CellTiter-Glo Luminescent Cell Viability Assay (Promega). Specifically, this was done by adding 90 μl of CellTiter-Glo reagent, shaking for two minutes to lyse the cells, incubating at room temperature for 10 minutes, and measuring luminescence (POLARstar OPTIMA).

### Propidium iodide (PI) stain cell cycle analysis

Cells were assayed for percentage of cells in each cell cycle phase or undergoing apoptosis (sub-G1 peak) by measuring DNA content following propidium iodide (PI) staining. Prior to staining, all media in the sample well was collected and placed in a 12 x 75 mm round bottom polystyrene tube (BD Falcon, 352054). Cells were washed once with PBS, the PBS was added to the polystyrene tube, and cells were subsequently treated with 0.25% trypsin for 5 minutes. Media was then used to resuspend the trypsin-treated cells, which were collected and placed in the polystyrene tube. Cells were pelleted by centrifugation for 5 minutes at 2800 RPM using an Immunofuge II. The media was aspirated, and the cells were fixed in 1 mL of ice cold 70% ethanol overnight at -20 °C. Cells were then warmed to room temperature (~15 minutes on bench), pelleted by centrifugation for 5 minutes, then rehydrated in 1 mL of room temperature PBS and incubated at 37°C for 15 minutes. Cells were then pelleted, the PBS aspirated, and the cells were resuspended in PI stain overnight. DNA staining was measured using a Becton-Dickinson FACSCalibur flow cytometer and analyzed using FlowJo Cell Cycle analysis to quantify the percentage of cells within the sub-G1, G1, S, or G2 phase.

### Carboxyfluorescein succinimidyl ester (CFSE) cell proliferation/division assay

Cells were stained with CFSE by resuspending 2 million HCT116 in 50 μM CFSE in PBS (1 mL total volume) in a 1.5 mL Eppendorf tube. Resuspended cells were incubated at 37 °C in the hot water bath for 20 minutes, washed once with 5 mL media, and resuspended in 13 mL media. Two mL of cells/media (approximately 300,000 cells/well) were added to each well on a 6-well plate on top of the siRNA transfection reagents as described previously. After 96 hours, cells were trypsin-treated, pelleted, and resuspended in PBS for flow cytometry analysis to measure CFSE staining. Remaining cells, after flow cytometry analysis, were used in western blot evaluation to confirm target gene depletion.

### Reagents

Poly-2-hydroxyethyl methacrylate (polyHEMA, P3932) and propidium iodide (PI, P4170) were purchased from Sigma-Aldrich. The ERK inhibitor SCH772984 was purchased from SelleckChem (S7101). CFSE dye was purchased from Tonbo Biosciences (10140–976 VWR). The Wee1 inhibitor MK-1775 (HY-10993) and CHK1 inhibitor AZD-7762 (HY-10992) were purchased from MedChem Express.

### Statistical analyses

*P* values were calculated using Prism Software (GraphPad, La Jolla, CA). A P value of less than 0.05 was considered statistically significant. Significance of TCGA RNASeq results were evaluated using an unpaired, two-sided t-test comparing the tumor tissue for each cancer type with the solid tissue normal samples from the same tissue. Significance of qPCR results was evaluated using one-way ANOVA with Dunnett’s post-test to individually compare all cell lines to the control cell line HCEC. The cell metabolic capacity assays, cell cycle analysis, and CFSE staining cell proliferation experiments were statistically evaluated using an unpaired, two-sided t-test to compare the effects of TIMELESS depletion to control cells that were transfected with non-targeting siRNA in each cell line or each cell line and each phase of the cell cycle. The significance of combination TIMELESS depletion and Wee1 or CHK1 inhibition was evaluated using a one-way ANOVA with Bonferroni’s Multiple Comparison test for the comparisons specified in the figure legend and were marked as being significant if the p value was less than 0.001. Data are shown as mean +/- standard deviation (SD) unless otherwise noted.

## Results

### TIMELESS is overexpressed in cancer

**Fig 1:**
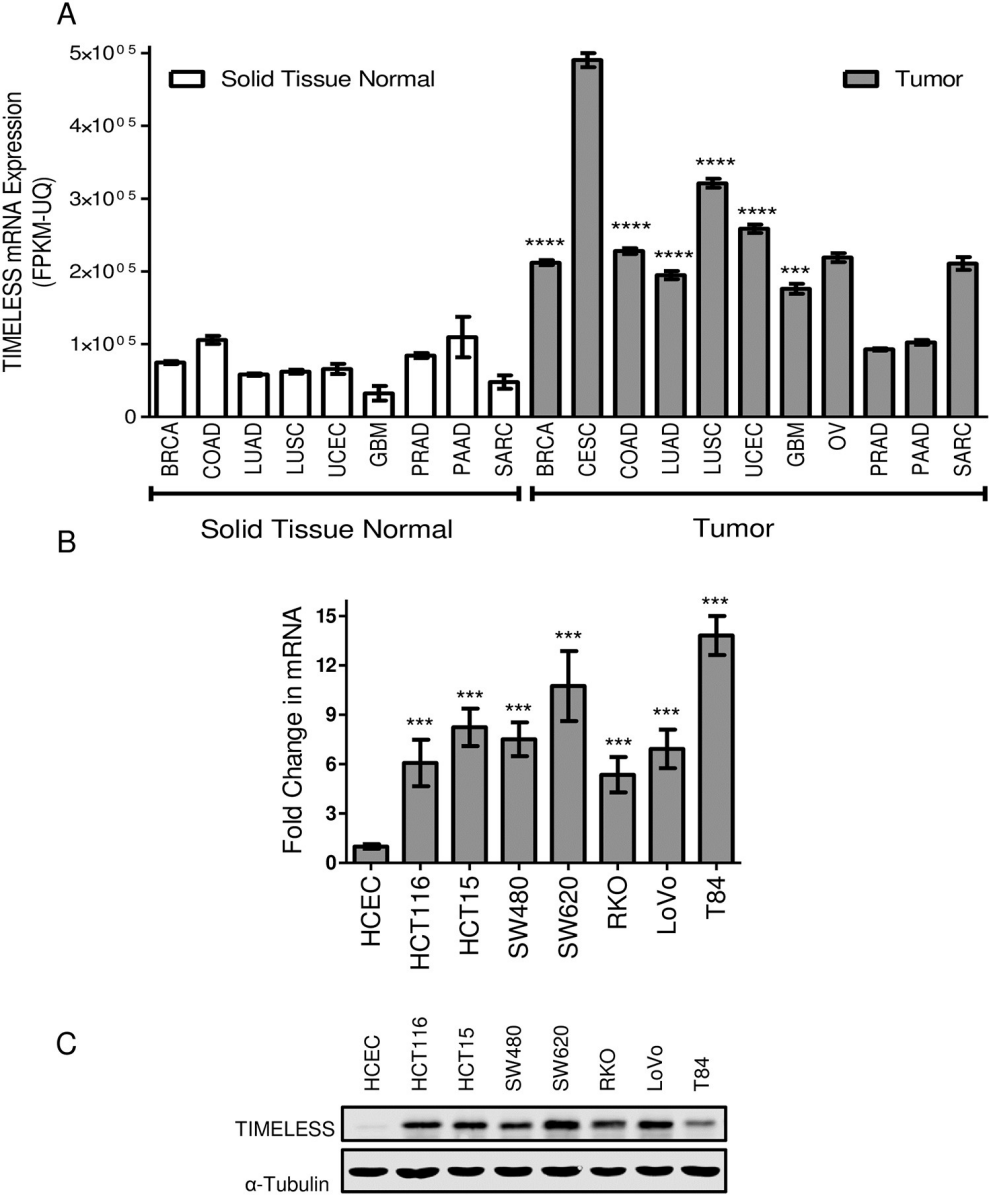
TIMELESS is overexpressed in cancer. (A) TIMELESS mRNA expression (RNASeq) data from TCGA for unpaired primary tumors and normal solid tissue samples. The results published here are in whole or part based upon data generated by the TCGA Research Network: http://cancergenome.nih.gov/. Data are shown as mean ± SEM. (B) RT-qPCR and (C) representative western blot of TIMELESS levels in a panel of colon tumor cell lines and immortalized, non-transformed HCECs. RT-qPCR data are shown as mean ± SD and represent three independent experiments. *** p < 0.001, **** p < 0.0001. Western blot in (C) is representative of five independent experiments comparing the expression of TIMELESS in HCECs to eight colon cancer cell lines as depicted.

TIMELESS is upregulated at the mRNA level in multiple tumor types compared to normal solid tissue (TCGA) (Fig 1A). In a panel of human colon cancer cell lines (HCT116, HCT15, SW480, SW620, RKO, LoVo, and T84), TIMELESS is upregulated at the mRNA (Fig 1B) and protein level (Fig 1C) compared to HCECs.

### ERK signaling promotes TIMELESS expression

**Fig 2:**
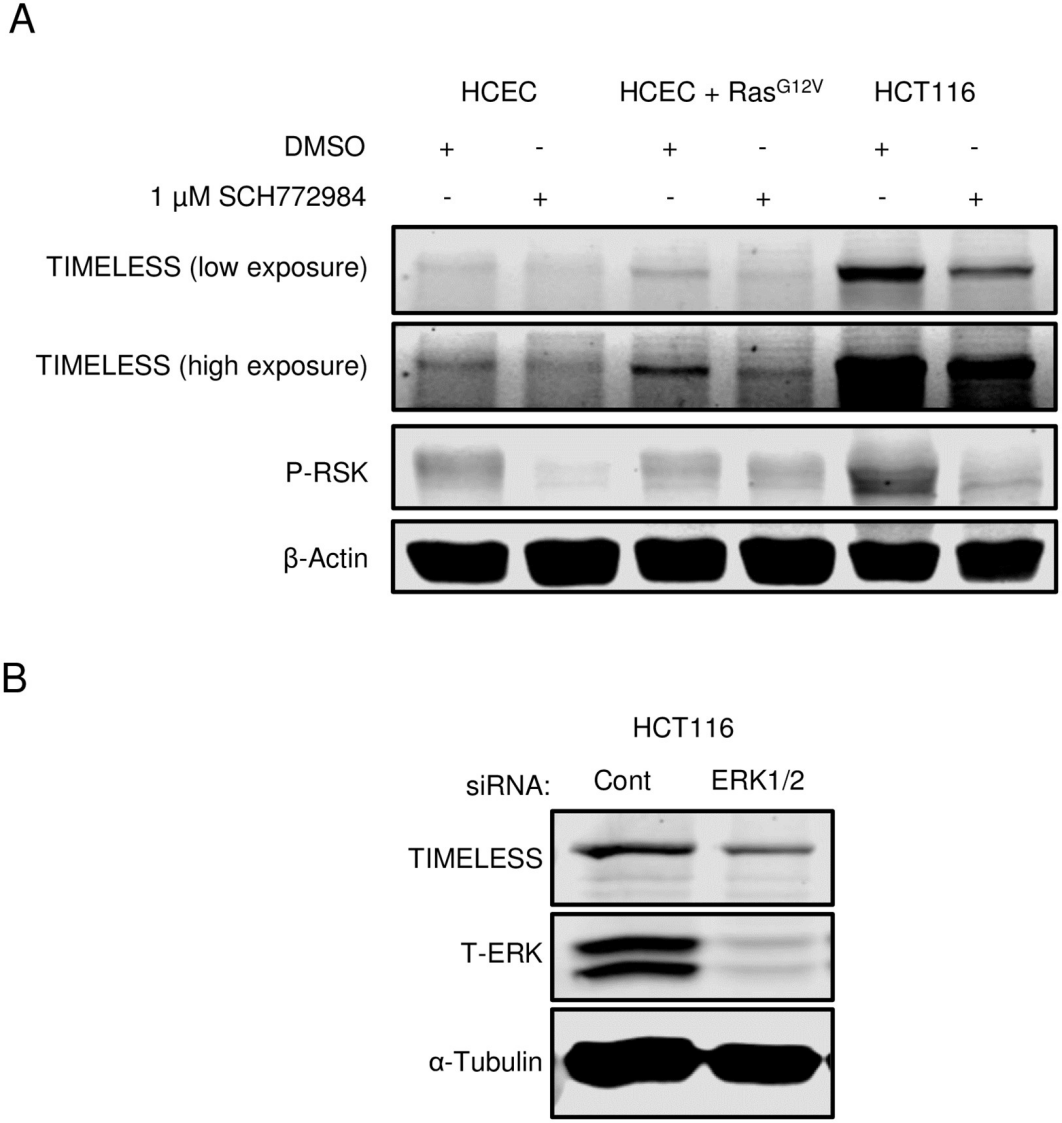
Activated ERK promotes TIMELESS expression. (A) Western blot of TIMELESS in HCECs, HCECs that stably express H-Ras^G12V^, and HCT116 colon cancer cells with DMSO or 1 μM SCH772984 (ERK inhibitor) treatment for 48 hours. (B) Western blot of TIMELESS following RNAi-mediated ERK depletion for 72 hours in HCT116 cells. Western blots shown in (A) and (B) are representative of three or more independent experiments.

To evaluate if oncogenic Ras contributes to increased TIMELESS expression in colon cancer, TIMELESS expression was examined in HCECs, HCECs that exogenously express mutant Ras (Ras^G12V^), and HCT116 cells with and without ERK inhibition. HCECs expressing exogenous Ras^G12V^ have increased TIMELESS expression relative to HCECs albeit not to the levels seen in the colon cancer cell lines tested. This increase in expression was abrogated with ERK inhibition with 1 μM SCH772984 (Fig 2A) demonstrating the vital role ERK activation plays downstream of activated Ras to promote TIMELESS expression. RSK phosphorylation is included as an indicator of ERK inhibition via SCH772984. In all three cell lines, RSK phosphorylation decreases by more than 50% by quantification following ERK inhibition. TIMELESS expression was dramatically reduced following ERK inhibition (Fig 2A), which was confirmed using RNAi-mediated ERK1/2 depletion in HCT116 cells (Fig 2B).

### TIMELESS depletion is detrimental to colon cancer cells

**Fig 3:**
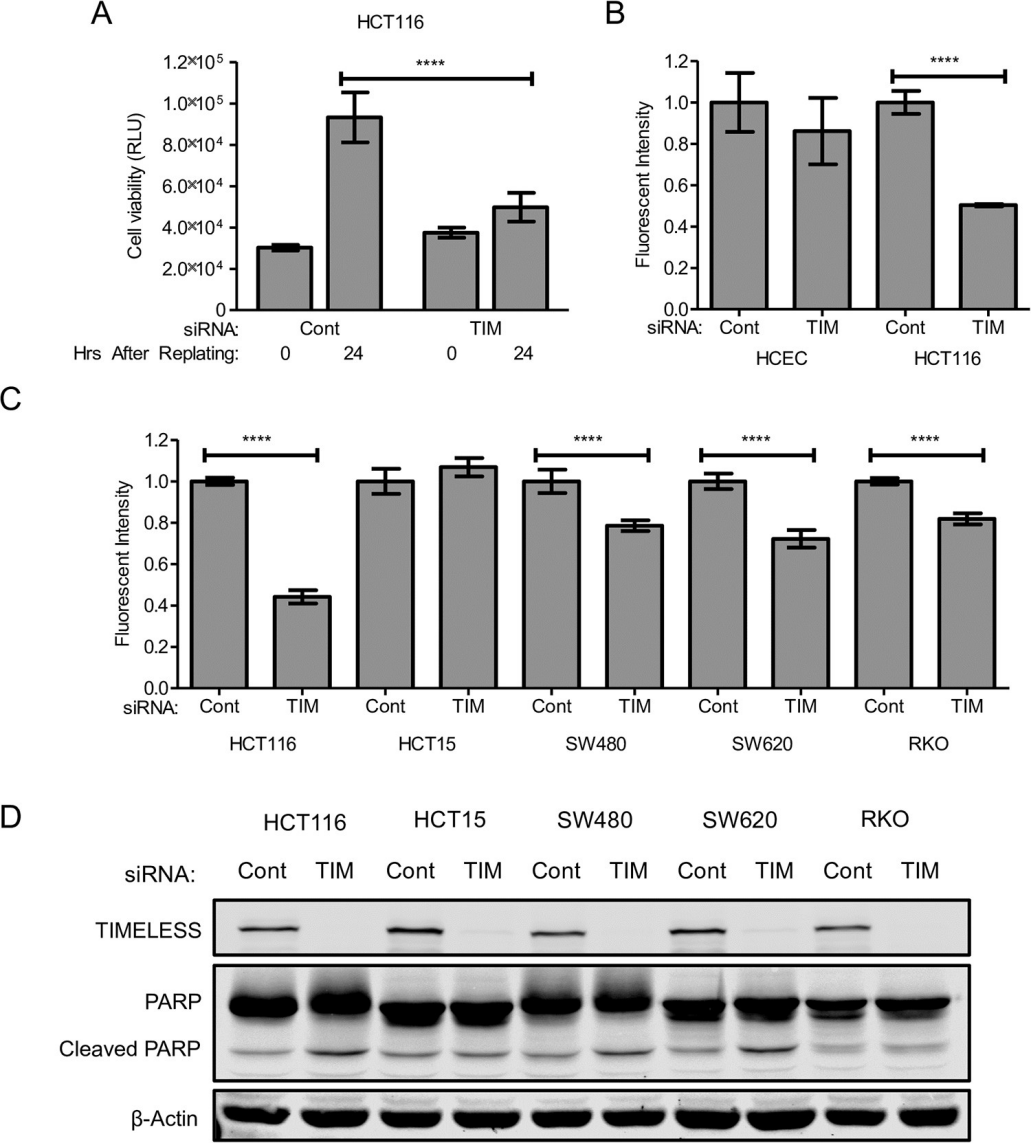
TIMELESS depletion is detrimental to colon cancer cells, but does not induce cell death. (A) Cell state was assessed in HCT116 colon cancer cells following RNAi-mediated depletion of TIMELESS that were replated on polyHEMA-coated plates 48 hours following transfection to simulate anchorage-independent conditions. Cell state was measured using CellTiter-Glo 0 and 24 hours after replating the cells. (B) Metabolic capacity of HCECs and HCT116 cells measured using alamarBlue following RNAi-mediated TIMELESS depletion for 72 hours in normal culture conditions. (C) Metabolic capacity was measured in a panel of colon cancer cells following RNAi-mediated depletion of TIMELESS using alamarBlue 96 hours after transfection. (D) Western blot of TIMELESS and PARP following RNAi-mediated TIMELESS depletion for 72 hours in HCT116, HCT15, SW480, SW620, and RKO colon cancer cells. Data are shown as mean ± SD and represent four independent experiments in (A), three independent experiments in (B), and five independent experiments in (C). **** p < 0.0001.

Initial biological validation of TIMELESS was performed by assessing the effect on cancer cells in anchorage-independent conditions following RNAi-mediated TIMELESS depletion by measuring cell growth on a polyHEMA-coated plate [41, 42] using CellTiter-Glo Luminescent Cell Viability Assay, as previously described [16]. Growth in anchorage-independent conditions was reduced substantially with TIMELESS depletion in HCT116 colon cancer cells (Fig 3A). Immortalized, yet non-transformed human colon epithelial cells (HCECs) [39] are unable to proliferate in an anchorage-independent environment and were therefore not assayed in anchorage-independent culture conditions. To compare the effects of TIMELESS depletion in HCT116 colon cancer cells to HCECs, RNAi-mediated depletion of TIMELESS was completed under normal plating conditions and cell metabolic capacity was measured using alamarBlue. TIMELESS depletion for 72 hours reduced cell metabolic capacity in HCT116 cells, but not HCECs (Fig 3B).

To determine the prevalence of the effects of TIMELESS depletion in colon cancer cells, cell metabolic capacity was measured in a panel of colon cancer cell lines following RNAi-mediated TIMELESS depletion for 96 hours using alamarBlue. TIMELESS depletion decreased the overall metabolic capacity by more than 20% in HCT116, SW480, SW620, and RKO colon cancer cells (Fig 3C). HCT15 colon cancer cells were not sensitive to TIMELESS depletion (Fig 3C). To determine if this decrease in overall metabolic capacity was a result of cells undergoing apoptosis versus a decrease in cell proliferation, PARP cleavage following TIMELESS depletion for 72 hours was assessed by western blot. TIMELESS depletion induced only a very slight increase in PARP cleavage in HCT116, SW480, and SW620 colon cancer cells and did not affect PARP cleavage in HCT15 or RKO cells (Fig 3D). This suggests the overall decrease seen in metabolic capacity of the cells is likely due to an overall decrease in cell number and cannot be attributed to an increase in apoptosis.

### TIMELESS depletion induces G2/M arrest

**Fig 4:**
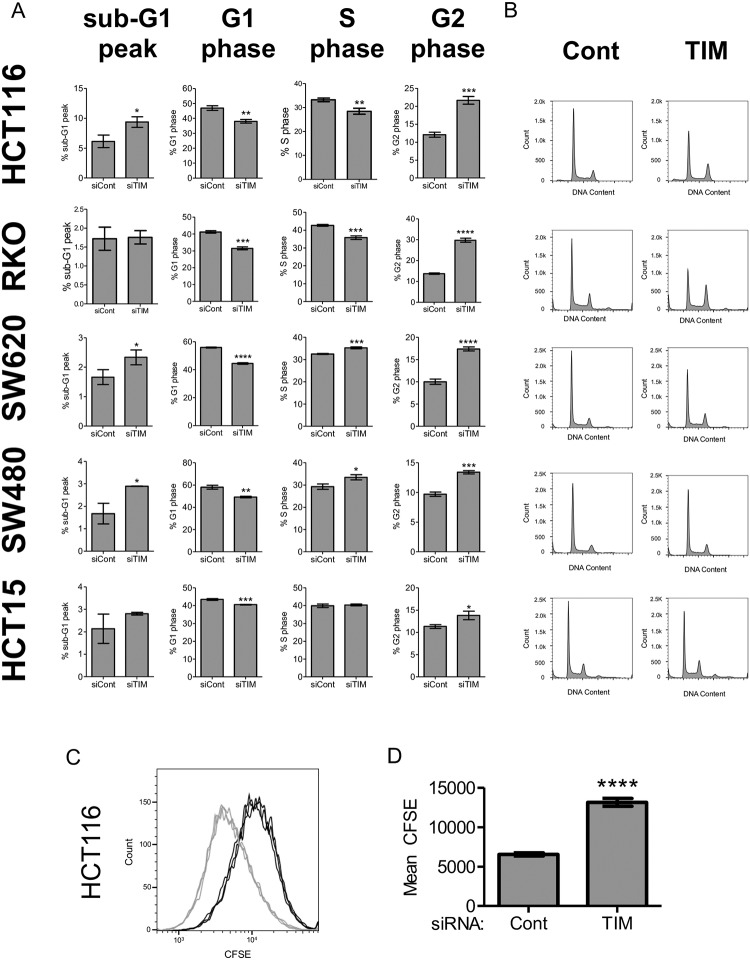
TIMELESS depletion induces G2/M arrest and decreases cell proliferation in colon cancer cells. (A) Quantification of the percent of cells in each phase of the cell cycle following RNAi-mediated TIMELESS depletion for 72 hours in HCT116, RKO, SW620, SW480 and HCT15 colon cancer cells from three independent experiments. Apoptosis (% of cells in the sub-G1 peak) and cell cycle were evaluated using propidium iodide staining followed by flow cytometry analysis. (B) Representative cell cycle histograms from (A). (C) Overlay histogram for three independent experiments of flow cytometry analysis of CFSE staining following RNAi-mediated TIMELESS depletion in CFSE-stained HCT116 cells for 96 hours. Control replicates are shown in gray, and TIMELESS-depleted replicates are shown in black. (D) Quantification of mean CFSE staining from (C). Data are shown as mean ± SD and represent three independent experiments in all cases. * p < 0.05, ** p < 0.01, *** p < 0.001, **** p < 0.0001.

TIMELESS depletion reduced metabolic capacity based on the alamarBlue viability assay in multiple colon cancer cell lines (Fig 3C); however, TIMELESS depletion did not significantly increase apoptosis based on very little, if any, increase in PARP cleavage (Fig 3D). Additionally, TIMELESS has been shown to play a role in triggering cell cycle checkpoints [23, 25, 28, 37, 38]. Therefore, cell cycle analysis was performed using propidium iodide staining and flow cytometry evaluation. In HCT116, SW620, and SW480 colon cancer cell lines a very small increase in sub-G1 peak was induced with TIMELESS depletion (Fig 4A). This is consistent with the very minor induction of PARP cleavage in these cell lines following TIMELESS depletion (Fig 3D). All five colon cancer cell lines underwent a decrease in percent of cells within G1 and an increase in percent of cells within the G2 phase (Fig 4).

### TIMELESS depletion reduces cancer cell proliferation

To confirm that TIMELESS depletion reduced cell proliferation, or specifically cell division, a carboxyfluorescein succinimidyl ester (CFSE) assay was completed. CFSE is a cell-permeable, fluorescent dye that covalently binds to intracellular molecules, particularly lysine and other amine-containing molecules. The covalent integration of CFSE is highly stable such that the fluorescence is sustained for long periods of time, and the dye is not leached to other cells. With each cell division, approximately half of the integrated CFSE is passed on to each daughter cell, such that the degree of CFSE staining can be used as a marker for cell division. Therefore, HCT116 cancer cells were stained with CFSE dye prior to RNAi-mediated TIMELESS depletion for 96 hours (S1 Fig). Flow cytometry analysis was then employed to measure CFSE fluorescence levels (Fig 4C). HCT116 cancer cells that lacked TIMELESS had increased mean levels of CFSE demonstrating they underwent fewer cell divisions (Fig 4D).

### G2/M arrest arises following TIMELESS depletion, which corresponds with an increase in γH2AX levels and subsequent phosphorylation of CHK1 and CDK1

One previous study demonstrated that TIMELESS supports cancer cells by increasing MYC expression and activity [12]. Another report proposed that TIMELESS could support cancer by supporting Ras signaling as Ras mRNA expression was downregulated following TIMELESS depletion [43]. Therefore, the potential for TIMELESS to support Ras or MYC to promote cell proliferation was evaluated by examining the effects of TIMELESS depletion on MYC expression and ERK phosphorylation/activation. RNAi-mediated TIMELESS depletion for 72 hours did not affect ERK phosphorylation or MYC protein levels in HCT116 cells (S2 Fig).

**Fig 5:**
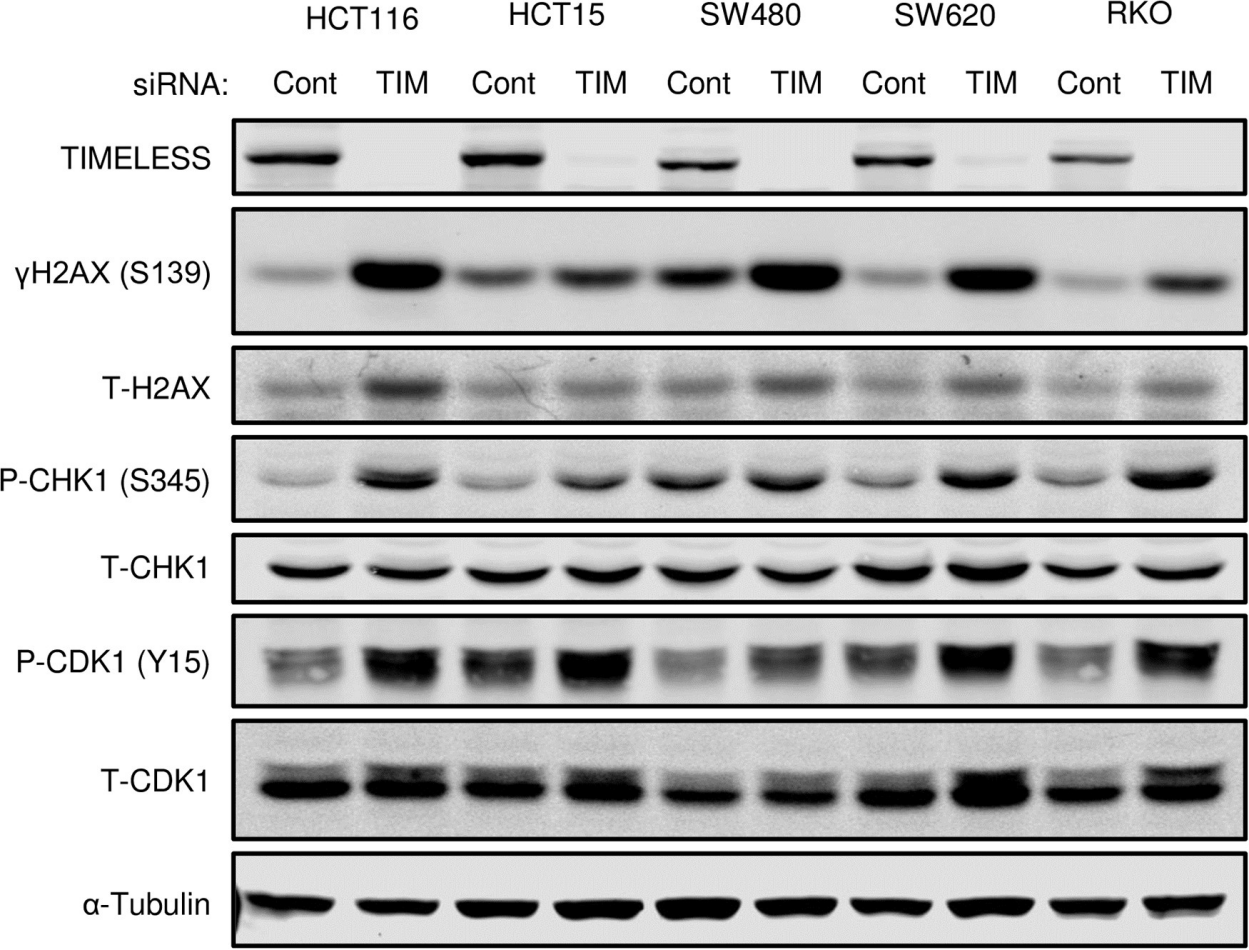
TIMELESS depletion induces G2/M arrest via CHK1 phosphorylation, which leads to CDK1 phosphorylation and inactivation. Western blot of phospho- and total-H2AX, phospho- and total-CHK1, and phospho- and total-CDK1 following RNAi-mediated TIMELESS depletion for 72 hours in a panel of colon cancer cells. The western blot shown is representative of three or more independent experiments in all five cell lines.

In normal cells, TIMELESS has been shown to promote DNA synthesis and DNA damage repair [21, 44]. In a panel of five colon cancer cell lines, RNAi-mediated TIMELESS depletion for 72 hours ubiquitously caused an increase in γH2AX, a marker of DNA damage (Fig 5); however, the cell lines demonstrated variable levels of H2AX phosphorylation with HCT15 cells resulting in a relatively small increase. Downstream of γH2AX, all five cancer cell lines demonstrated increased phosphorylation of CHK1 and CDK1 (Fig 5), which provides a mechanism for the G2/M arrest following TIMELESS depletion. The increase in γH2AX, P-CHK1, and P-CDK1 was recapitulated using four individual oligos targeting TIMELESS in HCT116 cells (S3 Fig). This mechanism appears to also be present in HCECs but is triggered to a lesser degree (S4 Fig) possibly as a result of intact DNA repair mechanisms, high fidelity DNA replication, and a slower cell proliferation rate in the normal HCECs compared to colon cancer cells. Previous studies have demonstrated HCT116 cells to have a doubling time of approximately 20–24 hours [45], while HCECs have a doubling time closer to 36–40 hours [39]. Additionally, overexpression of TIMELESS in HCECs demonstrates little effect on CHK1 or CDK1 phosphorylation (S5 Fig).

### Combination therapy with TIMELESS depletion and cell cycle checkpoint inhibitors

**Fig 6:**
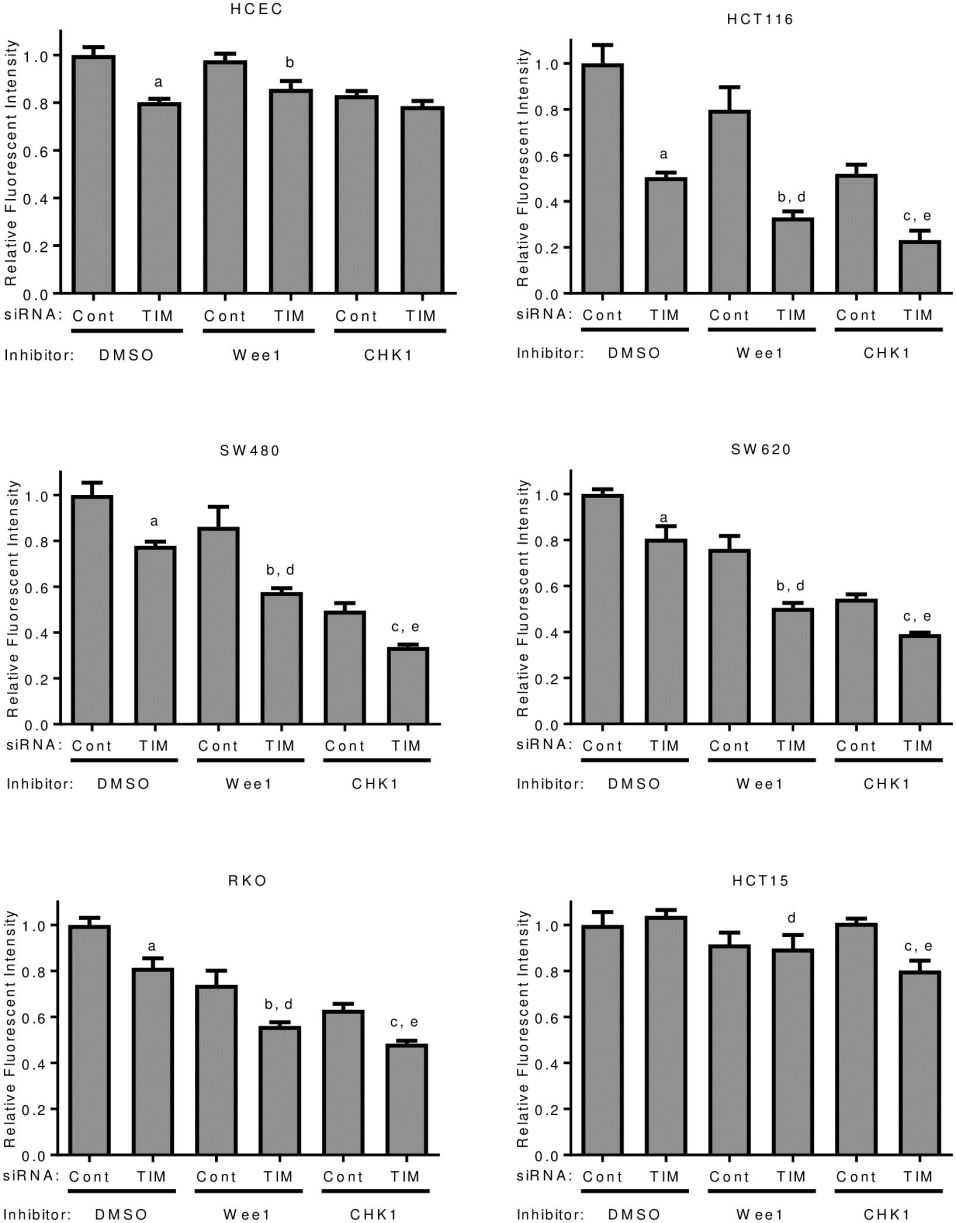
TIMELESS depletion sensitizes colon cancer cells to Wee1 and CHK1 inhibition. Cell metabolic capacity was measured in a panel of colon cancer cells following RNAi-mediated depletion of TIMELESS with Wee1 inhibition or CHK1 inhibition using alamarBlue assays 96 hours after transfection. 300 nM of Wee1 (MK-1775) or CHK1 (AZD7762) inhibitor was added 48 hours after transfection. Data are normalized to the DMSO treated control transfection (far left bar). Data are shown as mean ± SD and are representative of four or five independent experiments for each condition and cell line. The lower case letters denote a statistical significance (one-way ANOVA with Bonferroni’s Multiple Comparison test for the specified comparisons) with a p value less than 0.001 for the following comparisons: a—DMSO-treated siCont vs DMSO-treated siTIM; b—MK-1775-treated siCont vs MK-1775-treated siTIM; c—AZD7762-treated siCont vs AZD7762-treated siTIM; d—DMSO-treated siTIM vs MK-1775-treated siTIM; e—DMSO-treated siTIM vs AZD7762-treated siTIM.

Since TIMELESS depletion increases CHK1 phosphorylation and induces G2/M arrest, the potential for TIMELESS depletion to synergize with Wee1 or CHK1 inhibition was examined. With individual drug treatment, CHK1 inhibition was more lethal than Wee1 inhibition in all cell lines except HCT15 cells, which were more sensitive to the Wee1 inhibitor (Fig 6). Cell metabolic capacity was decreased with TIMELESS depletion in HCECs, but combination treatment with Wee1 or CHK1 inhibitors reduced this effect and overall TIMELESS depletion or CHK1/Wee1 inhibition had very little effect on these cells (Fig 6). In contrast, TIMELESS depletion in combination with Wee1 or CHK1 inhibition further decreased cell metabolic capacity than either perturbation alone in all the colon cancer cell lines tested except for HCT15 cells (Fig 6). Inhibition of Wee1 or CHK1 actually increased the percentage reduction in cell metabolic capacity following TIMELESS depletion suggesting at least additive effects with this combination in all of the colon cancer cell lines tested, but an inhibitory effect in HCECs. Interestingly, TIMELESS depletion has no effect in HCT15 cells with no drug treatment or Wee1 inhibition; however, CHK1 inhibition sensitized HCT15 cells to TIMELESS depletion.

## Discussion

These results demonstrate increased ERK activation promoted TIMELESS overexpression in cancer through a mechanism that is likely to be downstream of mutant Ras. This increase in TIMELESS expression promoted cancer cell proliferation as depletion of TIMELESS reduced the number of cell divisions due to cells undergoing G2/M arrest. This arrest corresponds with an increase in CDK1 inactivation mediated by phosphorylation of CDK1 downstream of CHK1 phosphorylation and activation in conjunction with an increase in γH2AX, a marker of DNA damage. However, it is still unclear if the damage is being caused because of the TIMELESS depletion or if the lack of TIMELESS is reducing the cells’ ability to repair and resolve naturally-occurring, endogenous damage from DNA replication.

This and previous studies have demonstrated that TIMELESS is highly expressed in multiple types of cancer [10–15]. One mechanism by which TIMELESS expression is increased is through increased ERK signaling (Fig 2). TIMELESS expression has been shown to vary with cell cycle, with the highest levels being seen in the S and G2 phases [25]. Therefore, it is possible that Ras and downstream ERK signaling promote TIMELESS expression as a side effect of increasing cell proliferation. It is also likely that TIMELESS expression is increased through other mechanisms since TIMELESS is overexpressed, at least at the mRNA level, in several cancers that are not commonly driven by oncogenic Ras including breast, uterine, ovarian, and cervical cancers (Fig 1). The TIMELESS promoter contains an E-box sequence such that CLOCK and BMAL1, the driving circadian rhythm transcription factors, are likely to promote the transcription of TIMELESS; however, this has not yet been demonstrated in mammalian cells. Additionally, the presence of this E-box opens the possibility that MYC could drive the expression of TIMELESS as MYC has recently been shown to disrupt the normal circadian-regulated expression of REV-ERBα and promote its constitutive expression by aberrantly binding to the E-box within its promoter [3].

The increase in TIMELESS expression in multiple cancers suggests that TIMELESS may ubiquitously promote cell proliferation by supporting high fidelity DNA synthesis, DNA damage repair, and cell cycle advancement potentially through numerous mechanisms as several possibilities have already been described [12, 19–21, 23, 24, 28, 38, 44, 46, 47]. Interestingly, TIMELESS has been shown to be required for the maintenance of cancer-associated viral genomes as well [48, 49], which could provide some rationale for its particularly high expression in cervical cancer that is almost always driven by human papillomavirus (HPV).

While TIMELESS depletion increased γH2AX and triggered the same downstream activation of CHK1 and inhibition of CDK1 in HCT15 cells as it did in the other cell lines examined (Fig 5), TIMELESS depletion did not reduce cell metabolic capacity in HCT15 cells (Fig 3). This could be due to dramatically disrupted and non-functioning cell cycle checkpoint function in these cells as HCT15 cells have a high basal level of P-CDK1 (Fig 5), yet maintain a high proliferative rate. HCT15 cells have an abundance of genetic alterations that disrupt DNA damage repair and cell cycle checkpoint pathways including mutations in ATM, ATR, BRCA1, BRCA2, CHK2, and FANCA. These or other alterations that promote cell cycle advancement in the presence of DNA damage could allow cells to lose their dependency on TIMELESS. Similarly, SW480 cells had a high basal level of P-CHK1 and demonstrated a limited capacity to phosphorylate and inactivate CDK1 even in response to a robust induction of γH2AX (Fig 5) suggesting these cells may also contain additional alterations that suppress DNA damage checkpoint signaling cascades and subsequent cell cycle arrest. Elucidating the mechanisms by which HCT15 cells promote cell cycle advancement even in the presence of phosphorylated and inactivated CDK1 and SW480 cells limit CDK1 inactivation by CHK1 may reveal novel mechanisms of resistance that could have therapeutic implications.

Despite the potential for some cancer cells to be insensitive or develop insensitivity to TIMELESS depletion, four of the colon cancer cell lines that were tested in this study demonstrated a substantial requirement for TIMELESS in order to maintain a high level of proliferation. G2/M arrest was particularly high in HCT116 and RKO cells, two colon cancer cell lines with wildtype p53. Several previous studies have demonstrated there are both p53-independent and p53-dependent pathways that contribute to cell cycle checkpoint activation in response to DNA damage. This suggests the p53-wildtype cells may trigger both p53-independent and p53-dependent pathways thereby inducing a more robust cell cycle arrest as compared to the mutant p53 cell lines (HCT15, SW480, and SW620) that only trigger p53-independent pathways. Additionally, combination therapy with TIMELESS depletion and either Wee1 or CHK1 inhibition demonstrated at least additive effects in four colon cancer cell lines, but not in HCECs (Fig 6), suggesting this combination may be an efficacious strategy for the treatment of colon cancer. The increased efficacy seen with the addition of the Wee1 or CHK1 inhibitor suggests that this combination could be inducing mitotic catastrophe in cancer cells. Interestingly, the CHK1 inhibitor AZD7762 did not decrease HCT15 metabolic capacity alone, but sensitized the HCT15 cells to TIMELESS depletion.

TIMELESS appears to have a highly conserved functional role in cells that could make it difficult to target therapeutically with a reasonable therapeutic index. However, HCECs expressed TIMELESS at lower levels and demonstrated less sensitivity to TIMELESS depletion. This could be a result of a slower proliferation rate [39], a difference that could also be exploited *in vivo* and that is the only means of cancer selectivity for several clinically approved chemotherapeutics. Targeting TIMELESS directly may be particularly efficacious in tumors with other defects in DNA damage repair pathways as they may be more dependent on TIMELESS to prevent or repair DNA damage. Recent studies have also demonstrated increased efficacy and decreased side effects when chemotherapeutics are dosed in a circadian fashion [4] suggesting a window of time exists when cancer cells, but not normal cells are more sensitive to chemotherapeutics. TIMELESS has been shown to be expressed in a circadian fashion in normal tissue; however, it is likely constitutively overexpressed in cancer due to its oncogene-driven expression. This opens the possibility that a circadian dosing scheme could increase the therapeutic index of TIMELESS inhibition.

This may not be necessary, however, as TIMELESS has previously been shown to physically interact with PARP at sites of DNA damage and PARP inhibitors trap TIMELESS with PARP at DNA lesions [26] effectively sequestering and possibly preventing TIMELESS from performing its other functions in the cell. Clinical trials with PARP inhibitors have demonstrated favorable side effect profiles such that these inhibitors are clinically approved for the treatment of multiple cancers, which provides optimism that, if developed, direct TIMELESS inhibitors may also be efficacious and have minimal side effects in patients. However, the effect of PARP inhibitors on TIMELESS has not been evaluated, and the high level of TIMELESS expression in cancer may mitigate any effect from sequestration of TIMELESS with PARP at sites of DNA damage. Additional work is needed to evaluate if PARP inhibitors functionally inhibit TIMELESS and if this contributes to their efficacy.

These data demonstrate that increased ERK activation promotes the overexpression of TIMELESS in HCT116 colon cancer cells. TIMELESS depletion increases γH2AX levels and phosphorylation of CHK1 and CDK1. This observation corresponded with a G2/M arrest suggesting that TIMELESS expression in colon cancer cells supports avoidance of cell cycle arrest to promote increased cell proliferation. These results demonstrate a possible role for TIMELESS in cancer and suggest that further examination of the link between circadian rhythm and cell cycle regulation may reveal novel approaches for the development of cancer therapeutics.

## Supporting information

S1 Table Sequences of qPCR primers. (PDF)

S2 Table Sequences of individual siRNA duplexes. (PDF)

S1 Fig Confirmation western blots of TIMELESS depletion and representative reference CFSE from the CFSE-staining cell proliferation experiment. (A) Western blot confirming TIMELESS depletion in all three biological replicates of CFSE-stained cells from Fig 4C–4D. (B) Overlay histogram for flow cytometry analysis of CFSE staining following RNAi-mediated TIMELESS depletion in CFSE-stained HCT116 for 96 hours. Control replicates are shown in gray, TIMELESS-depleted replicates are shown in black, and reference dye stain is shown on the far right. Western blot confirming TIMELESS depletion in all three biological replicates of CFSE-stained cells.
(PDF)

S2 Fig TIMELESS depletion does not affect ERK activation or MYC expression. Western blot of Myc, phospho-ERK, and phospho-MEK in HCT116 cells following RNAi-mediated TIMELESS depletion for 72 hours.
(PDF)

S3 Fig Individual oligos induce TIMELESS depletion, which causes increased γH2AX, CHK1 phosphorylation, and CDK1 phosphorylation in HCT116 cells. Western blot of phospho- and total-H2AX, phospho- and total CHK1, phospho- and total-CDK1 following RNAi-mediated TIMELESS depletion for 72 hours using four individual oligos or a pool of all four oligos in HCT116 cells.
(PDF)

S4 Fig TIMELESS depletion induces increased γH2AX, CHK1 phosphorylation, and CDK1 phosphorylation in HCT116 cells and to a lesser extent in HCECs. Western blot of phospho- and total-H2AX, phospho- and total-CHK1, phospho- and total-CDK1 following RNAi-mediated TIMELESS depletion for 72 hours in HCT116 and HCEC cells.
(PDF)

S5 Fig Exogenous TIMELESS expression has little effect on CHK1 phosphorylation and CDK1 phosphorylation in HCECs. Western blot of phospho- and total-CHK1, phospho- and total CDK1, and TIMELESS expression following exogenous TIMELESS expression for 48 hours in HCEC cells.
(PDF)

S1 File Raw western blot images: Fig 1C. (PDF)

S2 File Raw western blot images: Fig 2A. (PDF)

S3 File Raw western blot images: Fig 2B. (PDF)

S4 File Raw western blot images: Fig 3D. (PDF)

S5 File Raw western blot images: Fig 5. (PDF)
