# Supplementary material for: ERK-mediated TIMELESS expression suppresses G2/M arrest in colon cancer cells
Source: PLoS One. 2019 Jan 10;14(1):e0209224. doi: 10.1371/journal.pone.0209224 (PMC6328106; doi:10.1371/journal.pone.0209224)
Supplement: S1 Table — (PDF) [file pone.0209224.s001.pdf]

S1 Table. Sequences of qPCR primers

Target	Accession	Amplicon Length	Exons	Tm	F Primers 5' -> 3'	R primer 5' -> 3'
Human TIMELESS	NM_003920	120 bp	10-11	62°C	GAGACTTCTGCTCTGAGTTCC	CCAAGGCCCACATATAATAGGT
