# Supplementary material for: ERK-mediated TIMELESS expression suppresses G2/M arrest in colon cancer cells
Source: PLoS One. 2019 Jan 10;14(1):e0209224. doi: 10.1371/journal.pone.0209224 (PMC6328106; doi:10.1371/journal.pone.0209224)
Supplement: S2 Table — (PDF) [file pone.0209224.s002.pdf]

S2 Table. Sequences of individual siRNA duplexes

Target	Item #	siRNA	Target Sequence
Human TIMELESS	J-019488	5	UCAAUCGUCUGCUUAGUGA
		6	CAGGGUAGCUUAGUCCUUU
		7	GAGGGAGACACUUACCAUA
		8	CUACUGCUGGUCAGAAAUA
Human ERK1	J-003592	7	GACCGGAUGUUAACCUUUA
		8	CCUGCGACCUUAAGAUUUG
		9	CCAAUAAACGGAUCACAGU
		10	AGACUGACCUGUACAAGUU
Human ERK2	J-003555	11	UCGAGUAGCUAUCAAGAAA
		12	CACCAACCAUCGAGCAAAU
		13	GGUGUGCUCUGCUUAUGAU
		14	ACACCAACCUCUCGUACAU
Human Non-Targeting	D-001810	01	UGGUUUACAUGUCGACUAA
