# Supplementary figures and images for: ERK-mediated TIMELESS expression suppresses G2/M arrest in colon cancer cells
Source: PLoS One. 2019 Jan 10;14(1):e0209224. doi: 10.1371/journal.pone.0209224 (PMC6328106; doi:10.1371/journal.pone.0209224)

**S1 Fig**

**A**

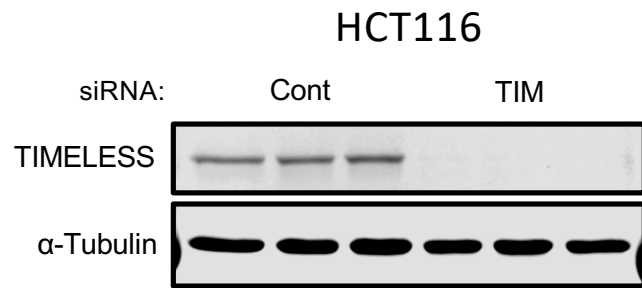

**B**

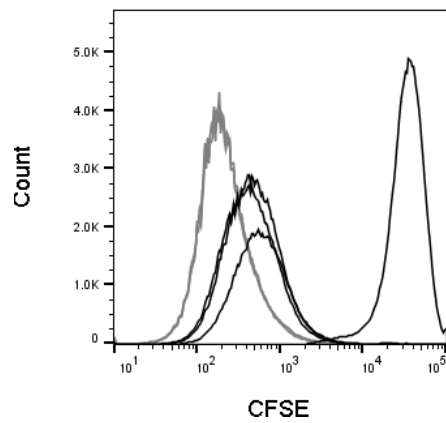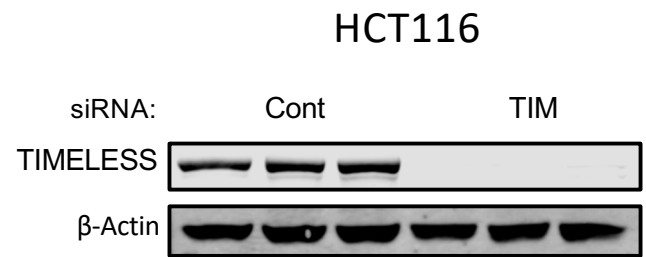

Supplement: S1 Fig — (A) Western blot confirming TIMELESS depletion in all three biological replicates of CFSE-stained cells from Fig 4C–4D. (B) Overlay histogram for flow cytometry analysis of CFSE staining following RNAi-mediated TIMELESS depletion in CFSE-stained HCT116 for 96 hours. Control replicates are shown in gray, TIMELESS-depleted replicates are shown in black, and reference dye stain is shown on the far right. Western blot confirming TIMELESS depletion in all three biological replicates of CFSE-stained cells. (PDF) [file pone.0209224.s003.pdf]

**S2 Fig**

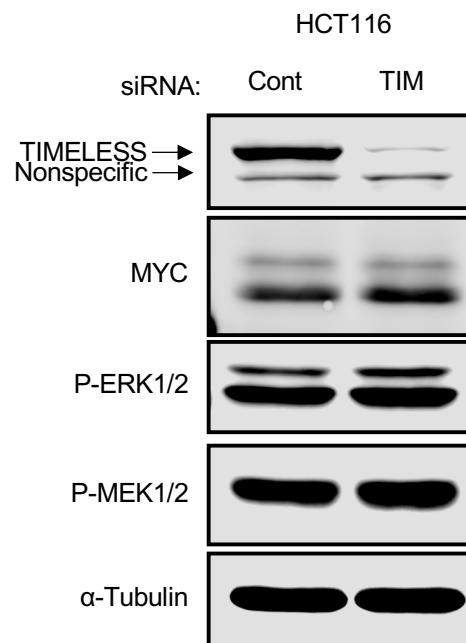

Supplement: S2 Fig — Western blot of Myc, phospho-ERK, and phospho-MEK in HCT116 cells following RNAi-mediated TIMELESS depletion for 72 hours. (PDF) [file pone.0209224.s004.pdf]

**S3 Fig**

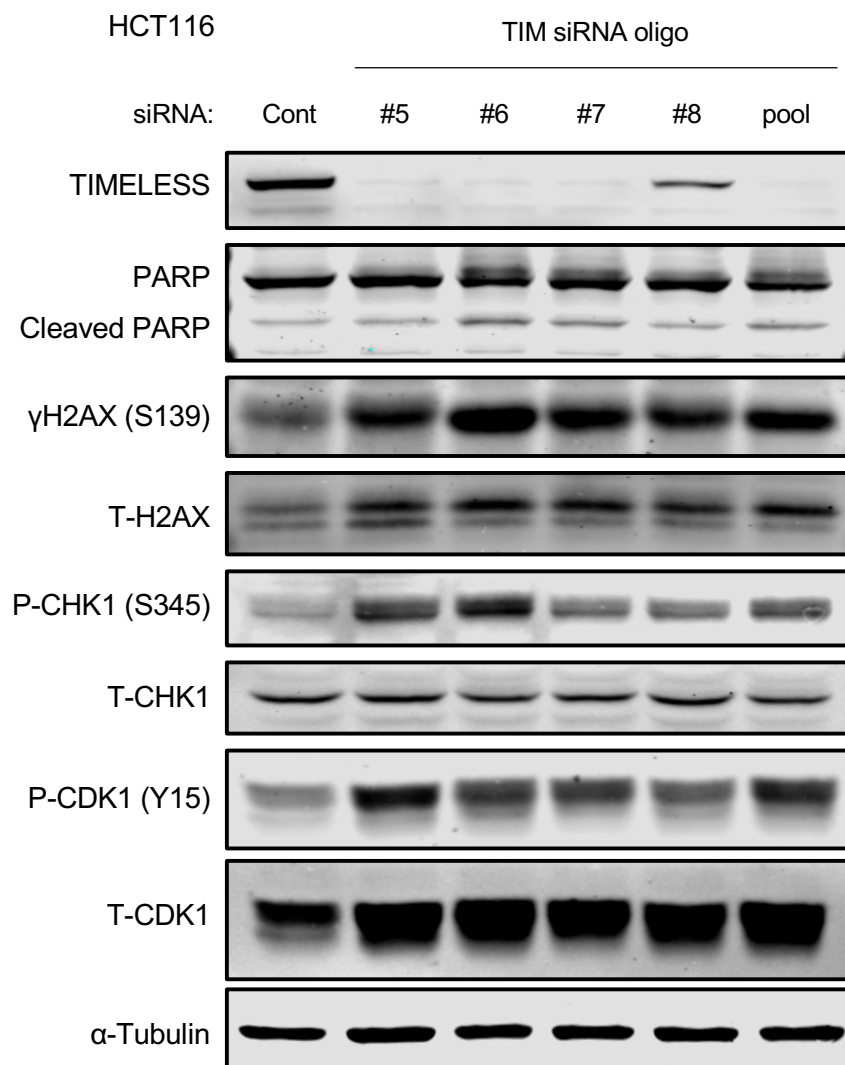

Supplement: S3 Fig — Western blot of phospho- and total-H2AX, phospho- and total CHK1, phospho- and total-CDK1 following RNAi-mediated TIMELESS depletion for 72 hours using four individual oligos or a pool of all four oligos in HCT116 cells. (PDF) [file pone.0209224.s005.pdf]

S4 Fig

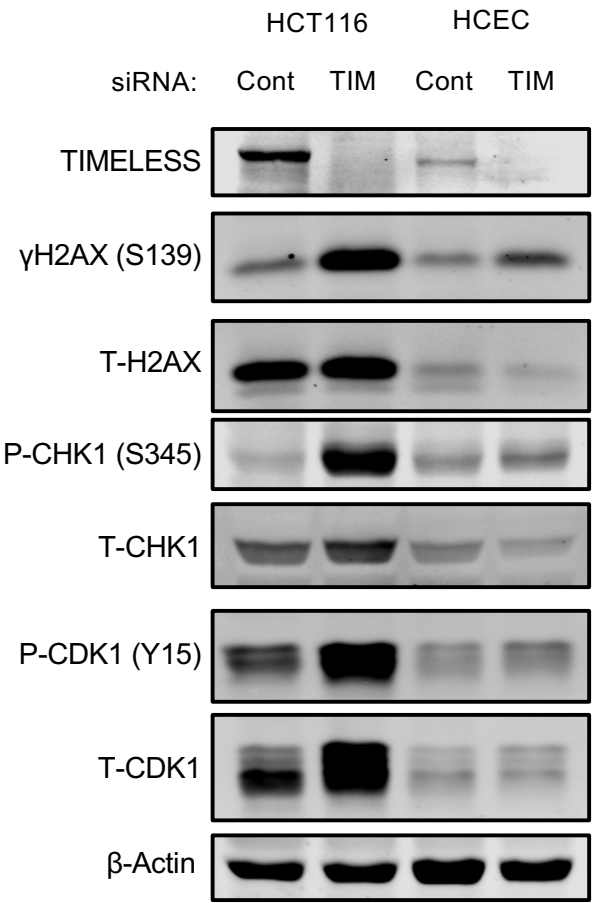

Supplement: S4 Fig — Western blot of phospho- and total-H2AX, phospho- and total-CHK1, phospho- and total-CDK1 following RNAi-mediated TIMELESS depletion for 72 hours in HCT116 and HCEC cells. (PDF) [file pone.0209224.s006.pdf]

**S5 Fig**

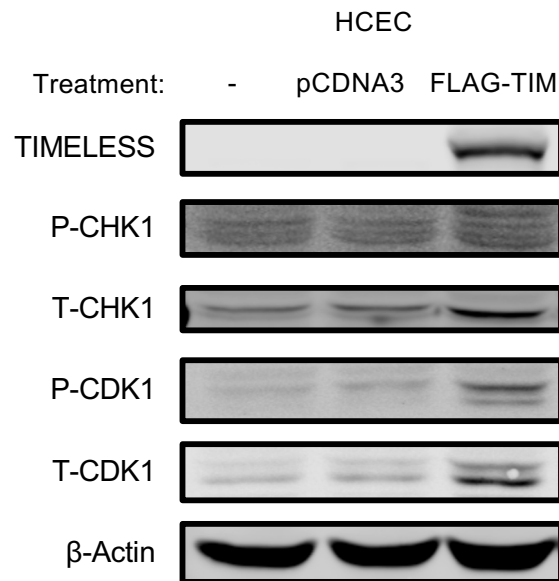

Supplement: S5 Fig — Western blot of phospho- and total-CHK1, phospho- and total CDK1, and TIMELESS expression following exogenous TIMELESS expression for 48 hours in HCEC cells. (PDF) [file pone.0209224.s007.pdf]

**Fig. 1C**

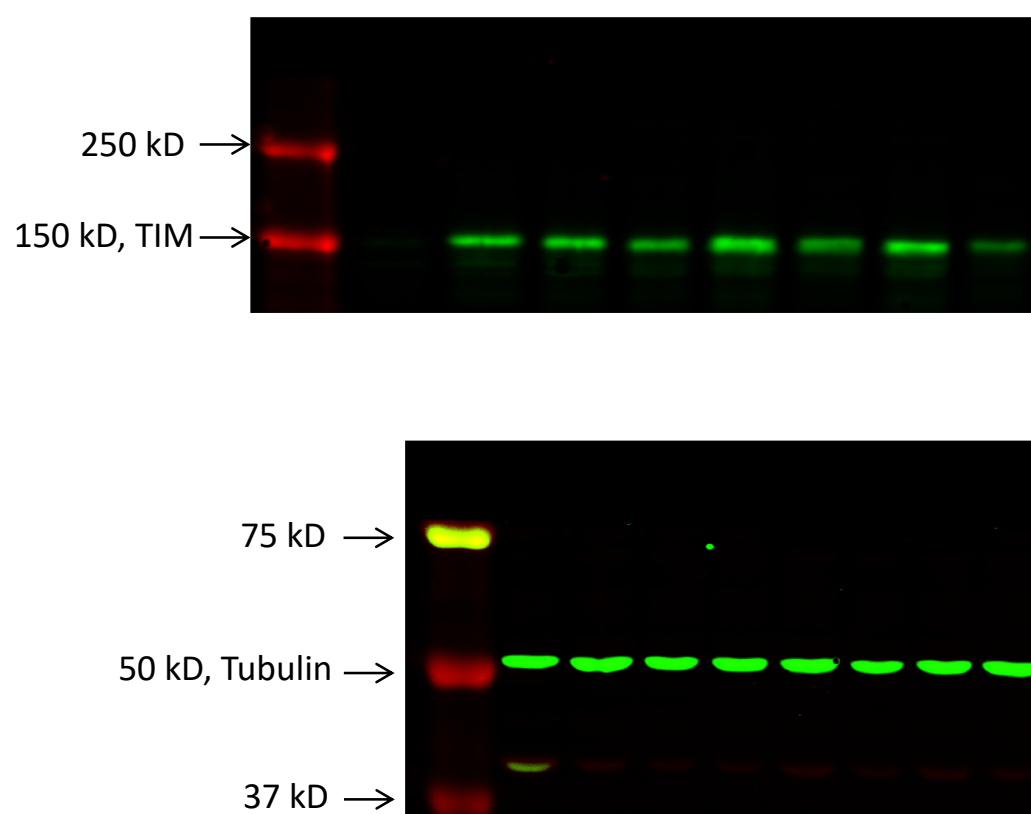

Supplement: S1 File — (PDF) [file pone.0209224.s008.pdf]

Fig. 2A

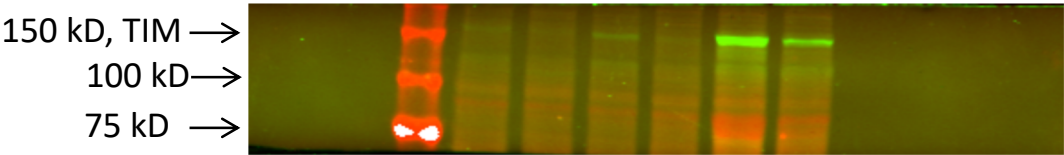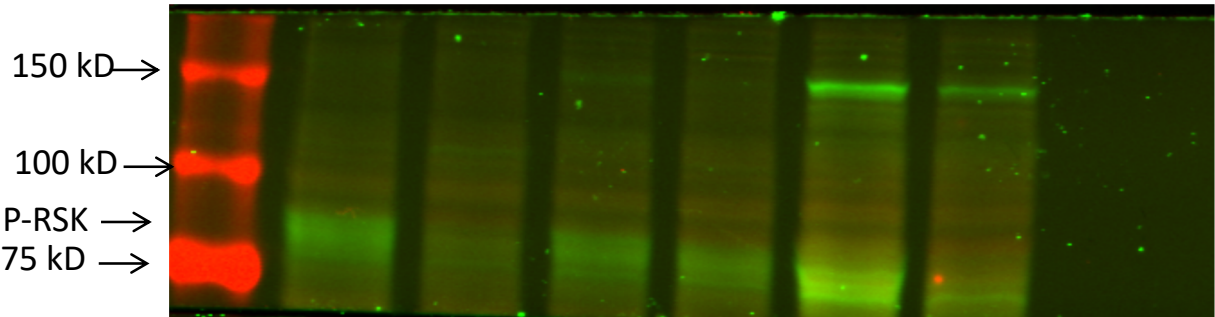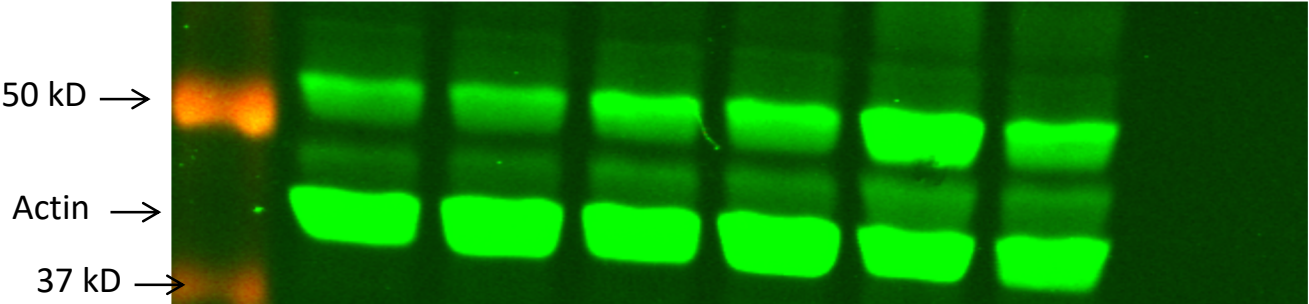

Supplement: S2 File — (PDF) [file pone.0209224.s009.pdf]

Fig. 2B

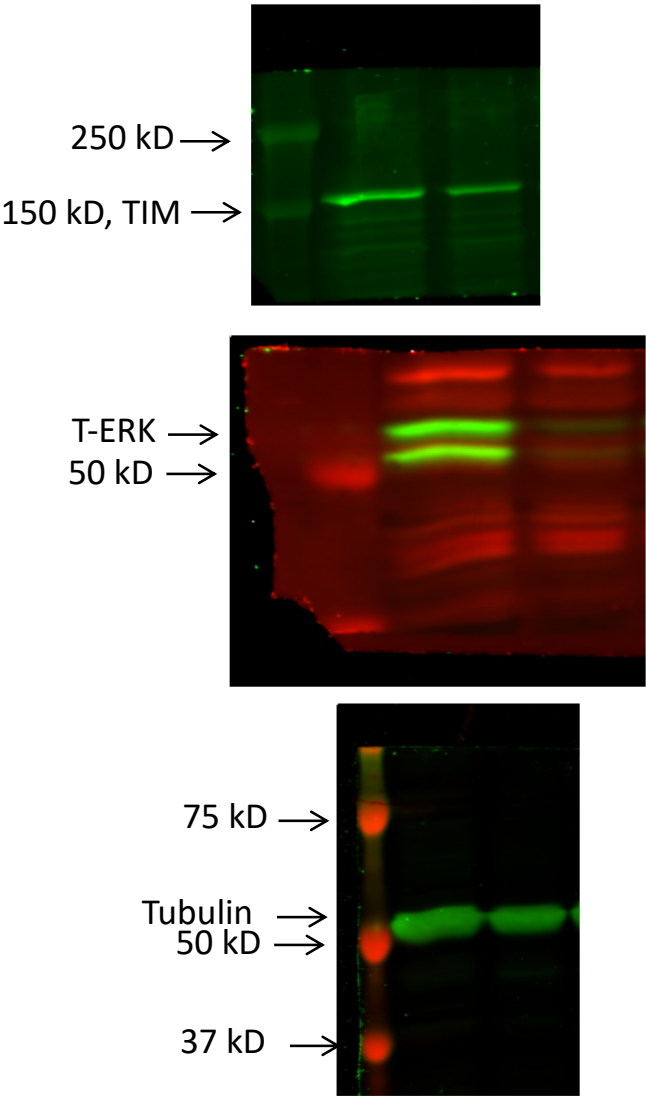

Supplement: S3 File — (PDF) [file pone.0209224.s010.pdf]

Fig. 3D

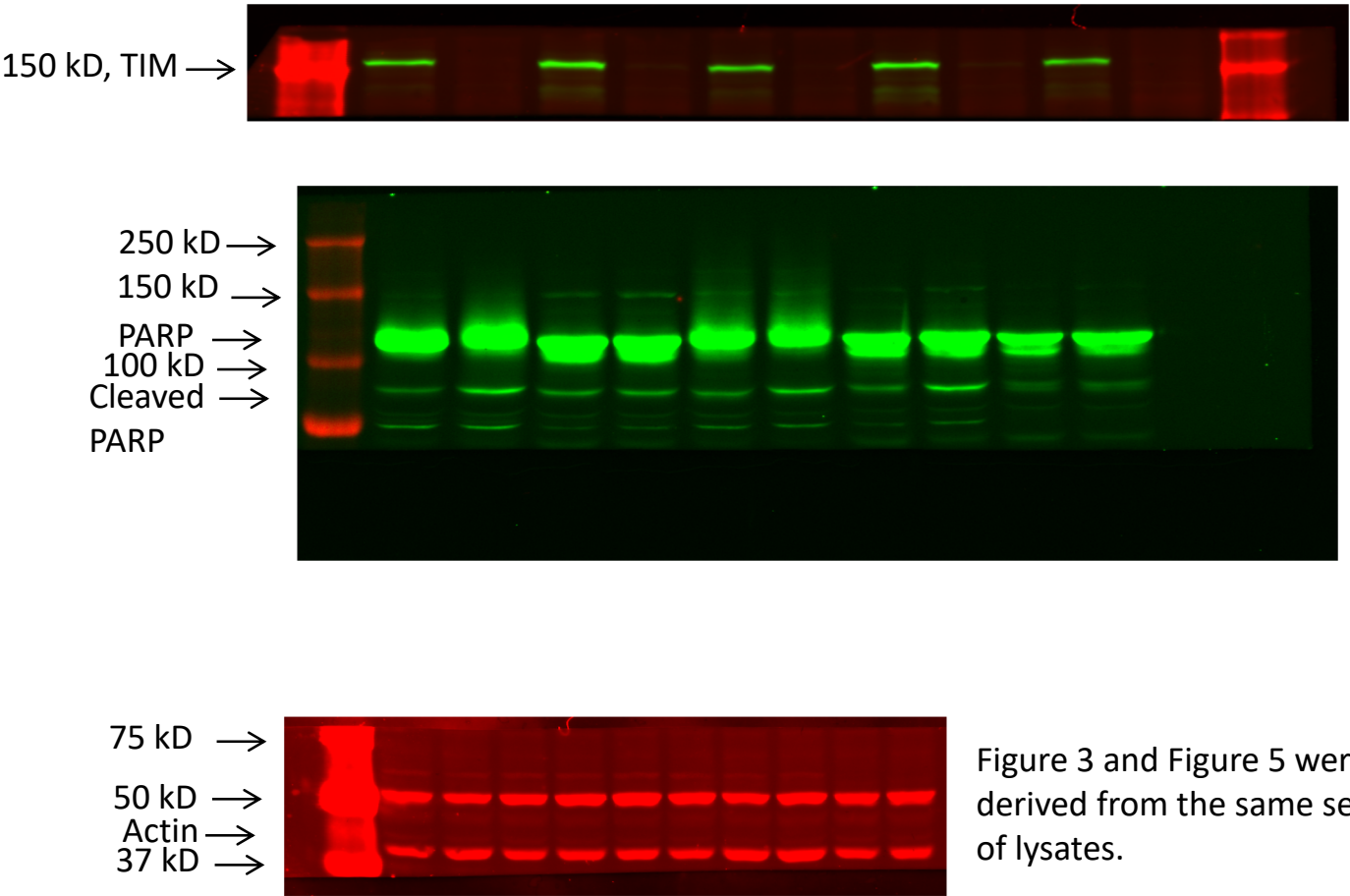

Figure 3 and Figure 5 were derived from the same set of lysates.

Supplement: S4 File — (PDF) [file pone.0209224.s011.pdf]

**Fig. 5**

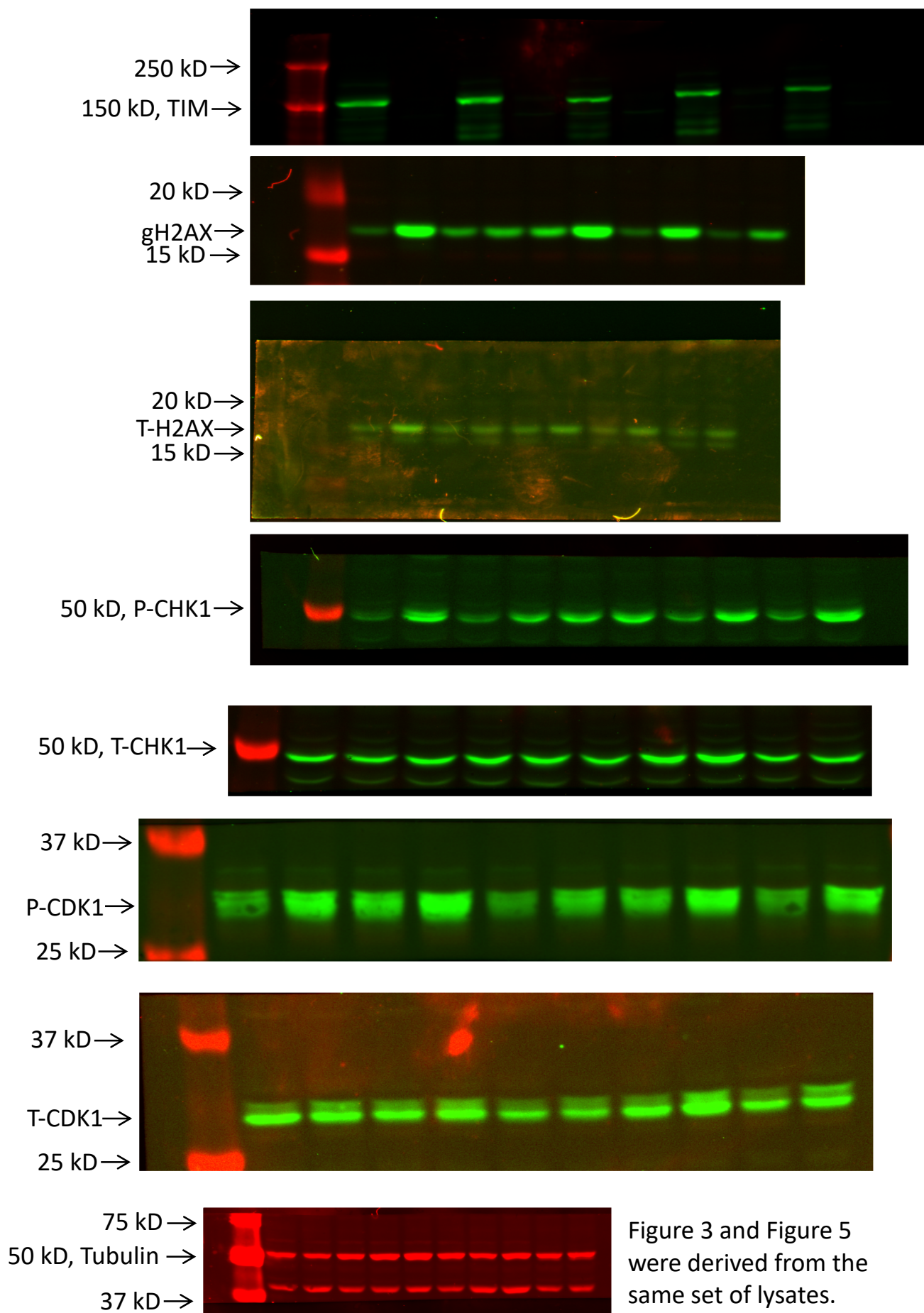

Supplement: S5 File — (PDF) [file pone.0209224.s012.pdf]
